# Correction to ‘Impaired nick recognition and ligation efficiency by LIG1 K845N variant linked to Huntington’s disease’

**DOI:** 10.1093/narmme/ugaf043

**Published:** 2025-12-17

This is a correction to: Jacob Ratcliffe, Camden M Lerner, Kanal Balu, Surajit Chatterjee, Kar Men Lee, Melike Caglayan, Impaired nick recognition and ligation efficiency by LIG1 K845N variant linked to Huntington’s disease, *NAR Molecular Medicine*, Volume 2, Issue 4, October 2025, ugaf038, https://doi.org/10.1093/narmme/ugaf038

The following changes have been made to the originally published manuscript. The name of the second co-author has been corrected now to read: "Camden M Lerner".

